# Vinpocetine, a Phosphodiesterase Type 1 Inhibitor, Mitigates Locomotor Hyperactivity in Female Mice Exposed to Lead During Development

**DOI:** 10.3390/brainsci15020150

**Published:** 2025-02-02

**Authors:** Ulisses C. Araujo, Fernanda Nunes, Bruno S. Gonçalves, Regina A. A. Gomes, Maria de Fátima R. Moreira, Andre Nunes-Freitas, Thomas E. Krahe, Yael de Abreu-Villaça, Alex C. Manhães, Cláudio C. Filgueiras

**Affiliations:** 1 Departamento de Ciências Fisiológicas, Universidade do Estado do Rio de Janeiro, Av. Prof. Manoel de Abreu 444, 5 andar, Vila Isabel, Rio de Janeiro 20550-170, RJ, Brazil; ulissescesar@yahoo.com.br (U.C.A.); bionanda82@yahoo.com.br (F.N.); biologobsg@gmail.com (B.S.G.); andrefreitas01@gmail.com (A.N.-F.); yael_a_v@yahoo.com.br (Y.d.A.-V.); ac_manhaes@yahoo.com.br (A.C.M.); 2 Centro de Estudos da Saúde do Trabalhador e Ecologia Humana (CESTEH), Escola Nacional de Saúde Pública, Rua Leopoldo Bulhões 1480, Manguinhos, Rio de Janeiro 21040-210, RJ, Brazil; reginaderne@gmail.com (R.A.A.G.); mfr.moreira55@gmail.com (M.d.F.R.M.); 3 Departamento de Psicologia, Pontifícia Universidade Católica do Rio de Janeiro, Rio de Janeiro 22451-000, RJ, Brazil; tekrahe@gmail.com

**Keywords:** locomotor hyperactivity, heavy metal, cyclic nucleotides, type 1 phosphodiesterase

## Abstract

Background/Objectives Studies in rodents indicate that disruptions in both cyclic adenosine monophosphate (cAMP) and cyclic guanosine monophosphate (cGMP) signaling pathways are involved in the development of hyperactive behavior. We examined whether vinpocetine, a phosphodiesterase type 1 inhibitor that enhances brain cAMP and cGMP levels, could mitigate locomotor hyperactivity in mice exposed to lead during early development. Methods Swiss mice were exposed to 90 ppm of lead in their drinking water throughout gestation and the first ten postnatal days. At postnatal day 10 (PN10), blood lead levels (BLLs) were about 30 µg/dL. At PN30, animals either received vinpocetine (20 mg/kg, i.p.) or a vehicle 4 h before the evaluation of locomotor activity in the open field. Results Lead-exposed males did not display differences in locomotor activity compared to controls, while lead-exposed females showed a significant increase in locomotion. Vinpocetine treatment significantly reversed the lead-induced hyperactivity in females. Conclusions These findings suggest that the cAMP and cGMP signaling pathways play a role in the hyperactivity induced by lead exposure.

## 1. Introduction

Although environmental lead levels have decreased significantly in recent decades, lead exposure continues to be a major public health concern [1]. Lead is a potent toxin, particularly harmful to the developing brain [1,2,3]. Its neurotoxicity involves mechanisms such as lead replacement for calcium, alterations in calcium homeostasis, the inhibition of N-methyl-d-aspartate receptors (NMDARs) [4], and an increase in oxidative stress [1,5]. These actions during critical development periods can cause long-lasting neurobehavioral impairments due to disruptions in essential neurobiological processes for brain growth and wiring [5]. Consequently, developmental lead exposure has been linked to several neurobehavioral disorders [1,3]. Of note, recent systematic reviews and meta-analyses have confirmed a strong association between low levels of lead exposure and behavioral symptoms of attention deficit hyperactivity disorder (ADHD) in school-aged children [5,6,7,8,9].

In rodents, lead exposure during gestation or the neonatal period causes molecular, cellular, and behavioral changes, helping to uncover the mechanisms of lead toxicity. In this regard, ambulation measured in open field tests is a useful tool. Lead-induced locomotor hyperactivity is associated with altered levels of dopamine, norepinephrine, and glutamate, all affecting brain excitability [4,10]. The balance between excitation and inhibition is crucial for precise brain function and plasticity [11,12], and its disruption is linked to neurodevelopmental disorders in humans and animal models, including autism and fetal alcohol spectrum disorder (FASD) [13,14]. Moreover, locomotor hyperactivity is observed in rodents with deletions of calcium-activated channels [15] or in those that were submitted to N-methyl-D-aspartate (NMDA) receptor blockade during development [16] or to an increase in oxidative stress [17], suggesting that the putative mechanisms of lead’s toxicity are capable of eliciting the increase in locomotor activity.

Even though the core symptoms of ADHD have been modeled in rodents [18], locomotor hyperactivity is the most frequently studied one. Locomotor hyperactivity has been largely described in rodents early exposed to lead [10,19,20]. Studies in rodent models of ADHD suggest that impairments in the cAMP and cGMP signaling pathways play a significant role in locomotor hyperactivity [21,22,23]. Developmental lead exposure reduces NR1 and NR2A subunits of NMDA receptors in the hippocampus of adolescent rats, lowers dopamine in the striatum and norepinephrine in the cerebellum [24], and disrupts downstream signaling, affecting cAMP-Response Element-Binding protein (CREB) binding, phosphorylation, and transcriptional activity [25,26]. This suggests that a weakened cAMP/cGMP signaling system may contribute to the hyperactive phenotype in lead-exposed animals.

Previous research shows that hyperactivity caused by developmental exposure to ethanol is mitigated by vinpocetine, a phosphodiesterase type 1 (PDE1) inhibitor [21,27]. The PDE1 inhibition by vinpocetine prevents the breakdown of cyclic nucleotides cAMP and cGMP to their 5′-monophosphates, maintaining the activation of protein kinases and transcription factors CREB and SRF [28,29]. These actions have been associated with the restoration of neuronal plasticity in the visual cortex of ferrets and mice [28,30], the improvement of learning deficits in rats in the Morris water maze [31], and the mitigation of rodent locomotor hyperactivity in the open field [21]. In addition, vinpocetine treatment reduced brain inflammation and oxidative stress in rats prenatally exposed to ethanol [27]. Considering that impairments in the cyclic nucleotide signaling system, inflammation, and oxidative stress may contribute to the locomotor hyperactivity observed in rodents early exposed to lead, this study aimed to test whether vinpocetine can restore normal locomotor activity in mice exposed to lead during development.

## 2. Materials and Methods

### 2.1. Animal Treatment

This study was approved by the ethics committee of the Universidade do Estado do Rio de Janeiro (protocol #: CEUA/040/2010) and conducted in compliance with Brazilian Law 11.794/2008. Swiss mice were bred and maintained in a temperature-controlled room (22 ± 1 °C) with a 12:12 h light/dark cycle (lights on: 2:00; lights off: 14:00). Access to food and water was unrestricted.

Lead exposure procedures were conducted as described previously [32]. The lead-exposed group (LEAD) consisted of mice whose dams (n = 9) were exposed to lead acetate trihydrate (90 ppm of lead) via drinking water from 60 days before mating until the 10th postnatal day (PN10). The control group (CONT) consisted of mice whose dams (n = 10) drank filtered water. At weaning (PN21), offspring from the same litter were separated by sex and housed in groups of 2–5 mice. To assess body mass gain over the first postnatal month, animals were weighed at PN2, PN10, PN21, and PN30. Ten lead-exposed and three control mice were randomly selected at the end of the exposure period (PN10) for the evaluation of blood lead levels (see detailed protocol below). The concentration of 90 ppm of lead was chosen because in a previous study [32], we demonstrated that the blood lead concentration (BLL) of adult mice that were exposed to this concentration for at least two months ranged from 4.4 to 16.6 µg/dL (mean = 9.6 ± 0.8). This BLL range is compatible with levels observed in studies of lead exposure in children [8,33,34] and with the blood lead reference value of 3.5 μg/dL proposed by the Centers for Disease Control and Prevention [35].

At PN30, animals from each litter were randomly allocated to receive either a single intraperitoneal dose of vinpocetine (VINP: 20 mg/kg) or an equal volume of vehicle. Vinpocetine was diluted in dimethyl sulfoxide, 0.5% *w*/*v*. Both drugs were purchased from Sigma-Aldrich (St. Louis, MO, USA). Behavioral testing was carried out 4 h after injections. Vinpocetine dose and waiting time interval were chosen based on previous works that demonstrated a 60% increase in cortical and hippocampal cAMP levels 4 h after a single IP injection of 20 mg/kg of vinpocetine and the mitigation of hyperactivity [21] and neuroplasticity deficits elicited by early ethanol exposure in rodents [31,36].

Following the above injection protocol, four experimental groups were defined: CONT+VEH (23 males and 19 females), CONT+VINP (22 males and 20 females), LEAD+VEH (24 males and 25 females), and LEAD+VINP (23 males and 29 females).

### 2.2. Open Field Test

Spontaneous locomotor activity was assessed in an open-field arena, following the methodology described in a previous study [21]. The open field arena consisted of a polypropylene box (37.6 × 30.4 × 17.0 cm) with the floor divided into 16 same-sized rectangles (7.6 × 9.4 cm)—2 in the periphery and 4 in the center. The experiments were performed during the dark phase of the daily cycle, 1–2 h after its onset. The animal was placed at the center of the arena, and its spontaneous locomotor activity was continuously recorded for 10 min using an overhead video camera. Following the testing session, the mouse was returned to its home cage, and the open-field arena was thoroughly cleaned before testing the next animal. An observer blind to treatment conditions performed behavioral quantification after the test conclusion.

The number of times the animal crossed the rectangles within the arena was used to quantify locomotor activity. For a crossing to be considered, a mouse had to place all four legs on a given rectangle. The following ambulation variables were evaluated: ambulation in the periphery (Pe, corresponding to the 12 rectangles adjacent to the walls), ambulation in the center (C, corresponding to the 4 rectangles in the center of the arena), C/Pe ratio, and total ambulation (Pe+C). Additionally, to address the difference in the number of rectangles between the center and the periphery, the number of rectangles crossed in the center was divided by 4 (C/4) and in the periphery by 12 (Pe/12) to enable accurate comparisons.

### 2.3. Determination of Blood Lead Levels (BLLs)

BLL procedures were carried out as previously described [32]. After completing behavioral tests, 23 randomly selected animals (lead-exposed: 6 males and 6 females; controls: 5 males and 6 females) were euthanized via cervical dislocation, and blood samples were collected through heart puncture. The blood samples were stored at −20 °C until BLL measurements were conducted, which occurred within 30 days of collection. The measurements were carried out using a Perkin Elmer 5100 atomic absorption spectrometer (Norwalk, CT, USA) with a graphite furnace, operating at a wavelength of 283.3 nm, atomization temperature of 1900 °C, and a detection limit of 0.6 µg/dL.

### 2.4. Statistical Analysis

To reduce litter effects and prevent over-sampling, data from both male and female animals within the same litter were averaged for each treatment group [37]. Separate univariate analyses of variance (uANOVAs) were conducted for litter size at PN2, body mass at PN30, BLL, and comparisons of C vs. Pe locomotor activity. Mixed-model analyses of variance (mANOVAs) were performed for both body mass and locomotor activity data. Exposure (LEAD or CONT), treatment (VINP or VEH), and sex were treated as between-subject factors. Age (PN2, PN10, and PN21) was considered as a within-subject factor for body mass gain and locomotor activity analyses. Lower-order ANOVAs were employed whenever significant interactions of treatment with other factors were detected. Individual group differences were assessed post hoc by Fisher’s Protected Least Significant Difference (FPLSD) tests. To simplify, we will report the results based exclusively on the averaged univariate F tests. Whenever the sphericity assumption was violated, the Greenhouse–Geisser correction, adjusting the degrees of freedom, was applied to avoid Type I errors. All data are presented as mean and ± SEM (unless otherwise mentioned). Significance was assumed at the level of *p* < 0.05, two-tailed.

## 3. Results

### 3.1. Blood Lead Levels

As depicted in Table 1, at PN10, the mean BLL for the LEAD group was significantly higher than that observed for the CONT group (uANOVA, F_(1,35)_ = 15.2, *p* < 0.001). As expected, the mean BLL measured at PN30 was significantly smaller than the PN10 value (uANOVA, F_(1,35)_ = 12.7, *p* < 0.001) and did not differ from the level observed in age-matched controls (exposure × age, F_(1,35)_ = 13.2, *p* < 0.001). Moreover, no sex difference in BLL at PN30 was found between males (LEAD group: 0.9 ± 0.3 µg/dL; CONT group: 1.3 ± 0.7 µg/dL) and females (LEAD group: 0.9 ± 0.3 µg/dL; CONT group: 1.1 ± 0.4 µg/dL).

### 3.2. Litter Size and Body Mass Gain

At PN2, the mean litter size of the CONT (10.5 ± 0.8 pups) and LEAD (12.9 ± 0.8 pups) groups did not differ.

**Figure 1:**
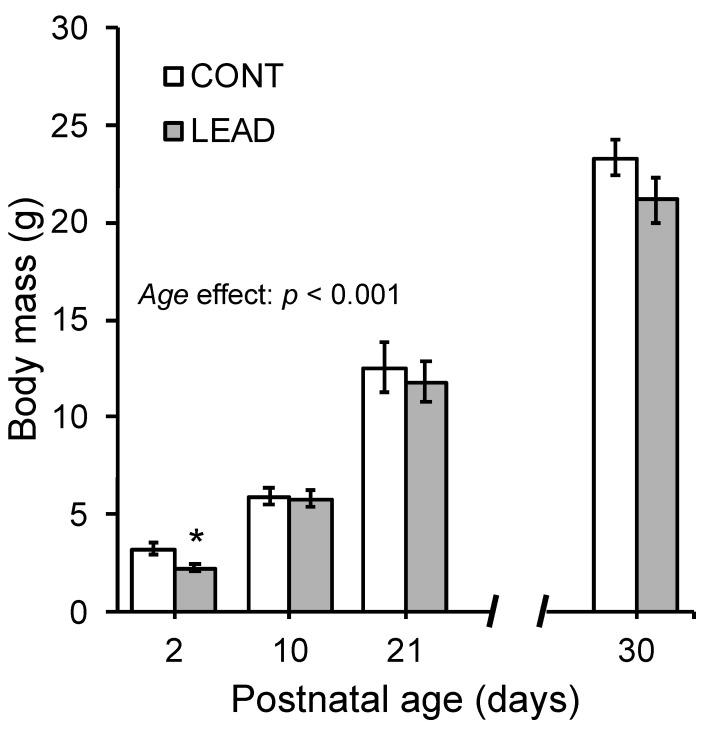
Mean body mass (± SEM) of mice exposed to lead during gestation and neonatal life (LEAD) or filtered water (CONT) at the second (PN2), tenth (PN10) and twenty-first (PN21) postnatal days. Note that lead exposure resulted in a significant body mass reduction at PN2. FPLSD: * *p* < 0.05.

Concerning body mass, as shown in Figure 1, the mean litter body mass from both experimental groups increased significantly from PN2 to PN21 (day effect: mANOVA, F_(1.1,15.6)_ = 109.2, *p* < 0.001). Although CONT litters were heavier than LEAD litters at PN2, no differences in body mass gain were observed between the experimental groups at PN10 and PN21. At PN30, the mean litter body mass of males (23.7 ± 1.0 g) was significantly higher (uANOVA, F_(1,34)_ = 4.4, *p* < 0.05) than that of females (20.1 ± 1.0 g). However, no differences were observed in litter body mass between CONT and LEAD, and there was no interaction between sex and exposure (Figure 1).

### 3.3. Open Field Test

**Figure 2:**
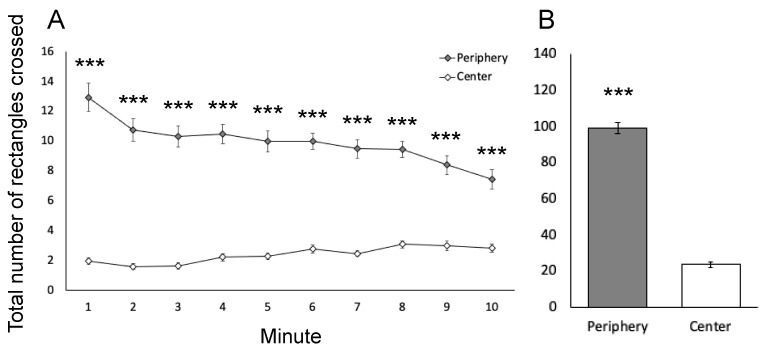
Ambulation in the periphery and in the center of open field throughout 1 min time intervals (**A**) and for the total 10 min test (**B**). Values represent mean (+S.E.M.) of the total sample. Note that the activity in the periphery was higher than that in the center. *** *p* < 0.001.

In line with previous findings [38], ambulation in the periphery of the open field was significantly greater than that in the center (uANOVA, F_(1,147)_ = 328.9, *p* < 0.001), regardless of sex and group assignment (Figure 2). This difference remained significant (uANOVA, F_(1,147)_ = 15.7, *p* < 0.001) even when ambulation was adjusted by the number of rectangles in the periphery (Pe/12: 8.3 ± 0.4) and center (C/4: 6.1 ± 0.4) of the arena. There were no significant main effects or interactions involving sex or group when comparing locomotor activity in the periphery and center of the open field.

**Figure 3:**
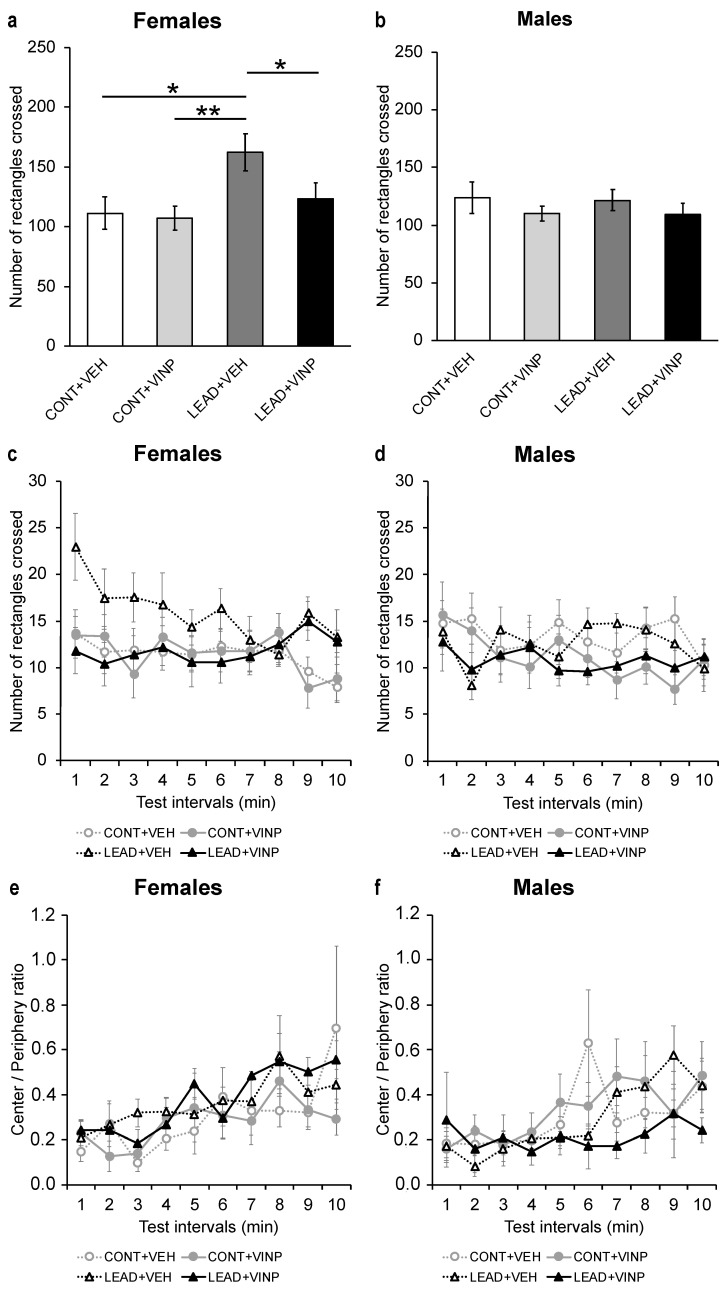
Mean (± SEM) of total ambulation (C+Pe) in the open field for PN30 (PN1 = day of birth) female (**a**) and male (**b**) mice exposed to lead during gestation and neonatal life (LEAD) or filtered water (CONT), treated with vinpocetine 20 mg/kg (VINP) or vehicle (DMSO) at PN30 (single i.p. dose 4 h prior to behavioral testing). Note that neonatal exposure to lead increased locomotor activity in females treated with the vehicle solution and that treatment with 20 mg/kg of vinpocetine restored locomotor activity to control levels. No differences were observed for males. In (**c**,**d**), total ambulation per 1 min interval is shown. The center/periphery ratio, a measure of anxiety-like behavior, for females (**e**) and males (**f**) was not affected by either lead exposure or vinpocetine treatment. FPLSD: * *p* < 0.05, ** *p* < 0.01.

Total ambulation (C+Pe) significantly decreased from the first (14.7 ± 1.0 rectangles) to the third (12.2 ± 0.9 rectangles) time interval for both the CONT and LEAD groups (mANOVA, time interval: F_(5.3,270.4)_ = 2.7, *p* < 0.05). Lead exposure during development induced locomotor hyperactivity in a sex-dependent manner (treatment × sex: rANOVA, F_(1,58)_ = 4.4; *p* < 0.05). The total ambulatory activity of lead-exposed females was approximately 31% higher (+50.8 rectangles) than that found for the control ones (Figure 3a,c). In contrast, developmental lead exposure did not lead to an increase in locomotor activity in males (Figure 3b,d).

Due to the treatment × sex interaction, subsequent statistical analyses were carried out separately for males and females. While vinpocetine treatment seemed to have no effect on the locomotor activity of lead-exposed males (Figure 3b,d), lead-exposed females that received vinpocetine displayed significantly less ambulatory activity (−23%, −38.5 rectangles) than those without treatment (Figure 3a,c). Moreover, the locomotor activity levels of lead-exposed females treated with vinpocetine were similar to those of both control groups, CONT+VEH and CONT+VINP (Figure 3a).

Since ambulation in the center is widely recognized as an indicator of anxiety [39], ambulation in the center was analyzed separately. For both the ambulation in the center (mANOVA, time interval: F_(6.3,326.3)_ = 7.9, *p* < 0.001) and the C/Pe ratio data (mANOVA, time interval: F_(1.5,12.3)_ = 4.7, *p* < 0.05; see Figure 3e,f), increases in values were observed across the 10 time intervals. However, no differences were found between the groups for these two variables. Taken together, these results suggest that lead exposure during development did not affect anxiety. Additionally, the treatment with vinpocetine did not differentially impact anxiety levels.

## 4. Discussion

The present study shows that lead exposure during development increases locomotor activity in females. The BLL is compatible with levels described in environmentally exposed humans [40]. Treatment with vinpocetine significantly ameliorated this lead-induced hyperactivity. It is important to highlight that vinpocetine treatment was administered long after the lead exposure period, during a developmental stage comparable to infancy/adolescence in humans. These findings suggest that cyclic nucleotide signaling systems, particularly cAMP and cGMP, play a role in lead-induced hyperactivity. Furthermore, the results support the potential therapeutic use of vinpocetine in individuals who were exposed to lead during development.

### 4.1. Sex Difference in Lead-Induced Locomotor Hyperactivity

The finding that only female mice exhibited lead-induced locomotor hyperactivity contrasts with studies demonstrating that early exposure to lead induces marked locomotor hyperactivity in both males and females [10,19,20,41,42,43,44,45]. However, it aligns with other rodent studies showing that some effects of developmental lead exposure manifest differently in males and females [46,47,48,49,50]. This discrepancy may be associated with the lower level of lead exposure in the present study compared to the levels typically used to investigate the neurobehavioral consequences of developmental lead exposure. In fact, juvenile/adolescent locomotor hyperactivity was described in offspring whose dams received at least 500 ppm of lead in drinking water during pregnancy and lactation [10,19,20,42,43,45]. However, a lower level of lead exposure (150 ppm in drinking water) from gestation to weaning reduced dopamine levels in the female offspring’s striatum and prefrontal cortex but did not affect their male siblings [48]. Similarly, while no sex differences were observed at higher doses of lead, females that received 50 mg of lead via drinking water throughout pregnancy and lactation until weaning presented more pronounced anxiety-like behavior and impairment in long-term memory [51]. Some evidence suggests that lead exposure during development leads to a series of sex-dependent epigenetic changes that influence gene expression patterns, which in turn can affect the development of the nervous system [51,52,53]. In general, the epigenetic consequences of developmental lead exposure were more pronounced in females, and the results were present long after cessation of exposure [51,52,54]. Taken together, these data suggested that, at the level of lead exposure used in the present study, female offspring were more susceptible to the neurotoxic effects of lead than males.

Apart from the level of exposure, some neurobehavioral outcomes of developmental lead exposure, such as locomotor hyperactivity, depend on the age at which animals are evaluated [19,50,55,56,57]. For instance, male rats exposed to lead (250 ppm in drinking water) during development exhibited increased locomotor activity in an open-field test on postnatal day 23 but not on postnatal day 70 [19]. No difference in locomotor activity was observed in 12-week-old male rats exposed to 250 ppm lead during pregnancy [58]. Similarly, rodents that are early exposed to other neurotoxic agents, such as ethanol, are hyperactive when juvenile [21,59,60,61], but mature subjects are not different from controls [62,63,64]. Interestingly, in diseases with a hyperactive component, such as ADHD, both hyperactivity and attention deficits decline with age [65], with hyperactivity symptoms decreasing at higher rates than inattention symptoms [66]. While boys outnumber girls during infancy, in adolescence/young adulthood, gender ratios tend to be equal [67]. Despite this, females demonstrate greater stability in comorbid psychopathology from childhood into adolescence than males [68]. Based on these data, it is not unreasonable to suppose that the absence of locomotor hyperactivity in males could reflect a sex difference in velocity/efficiency in which compensatory mechanisms operate in the brains of developmentally lead-exposed animals.

### 4.2. Vinpocetine Ameliorates Lead-Induced Locomotor Hyperactivity

Studies conducted in both humans with ADHD and rodents have suggested that a hypofunctional catecholaminergic system plays a major role in the manifestation of the hyperactivity phenotype [69,70]. Accordingly, agents that enhance catecholaminergic activity, such as methylphenidate and amphetamine, can mitigate locomotor hyperactivity in rodents exposed to lead during development [71]. It is noteworthy that the protective potential of vinpocetine has been associated with its ability as a phosphodiesterase inhibitor to alter extracellular dopamine levels; however, the results are not consistent [72,73,74]. In primary cultured rat mesencephalic neurons, the administration of vinpocetine decreased the intracellular dopamine level and increased the extracellular dopamine and 3,4-Dihydroxyphenylacetic acid (DOPAC) levels [74]. In contrast, in striatal isolated nerve endings, the presence of vinpocetine decreased internal dopamine and increased DOPAC but did not change the baseline release of dopamine [72,73].

Under physiological conditions, the activation of dopaminergic receptors stimulates cAMP synthesis through an increase in adenylyl cyclase activity and, via an upregulation in neuronal nitric oxide synthase activity, leads to cGMP production [75]. It has been suggested that a dopamine deficit would result in the reduced stimulation of dopamine receptors and a decrease in cAMP and cGMP synthesis [39]. In this context, a growing body of evidence suggests that impairments in the cyclic nucleotide cAMP and cGMP signaling pathways underlie locomotor hyperactivity [21,22,23,76]. In rodents, locomotor hyperactivity is observed after the deletion of serum response factor (SRF) in dopaminoceptive neurons [77] and cAMP-dependent protein kinase (PKA) inhibition [22]. Guanylyl cyclase-C knockout mice exhibited locomotor hyperactivity and attention deficits, which were reversed by amphetamine and the acute activation of cGMP-dependent protein kinase (PKG) [78]. Additionally, both the reduced levels of phosphorylated CREB in rats exposed to lead during development [25] and the observation that a low concentration of lead stimulates calmodulin-dependent phosphodiesterase activity in vitro [79] suggest that cAMP and cGMP signaling pathways are weakened in animals exposed to lead during development.

Intracellular levels of cAMP and cGMP are determined by the balance between its synthesis and breakdown. While cAMP is synthesized from adenosine triphosphate by adenylyl cyclase, cGMP is synthesized from guanosine triphosphate by guanylate cyclase [80]. Both cAMP and cGMP are broken down by PDEs [81]. Therefore, the inhibition of PDE by vinpocetine promote a quick increase cAMP and cGMP levels [21,28]. The vinpocetine-mediated amelioration of neurobehavioral deficits caused by early exposure to neurotoxic agents such as ethanol is associated with the restoration of cAMP and cGMP levels in ferrets [28], rats [31], and mice [21,30]. Importantly, vinpocetine also enhances the phosphorylation of transcription factors like CREB, SRF, and erythroblast transformation specific transcription factor Like-1 (ELK1) [29]. The PDE1 family comprises three isoforms: PDE1A, PDE1B, and PDE1C [29]. PDE1A and PDE1B, constituting at least 90% of total brain PDE1 activity, are expressed in brain regions typically linked to locomotor hyperactivity. Specifically, PDE1A exhibits high levels of expression in the cerebral cortex and hippocampus, while PDE1B is primarily expressed in dopaminergic regions, including the striatum and nucleus accumbens. PDE1C, on the other hand, is expressed in the olfactory neuroepithelium, substantia nigra, and thalamus [29]. Based on the aforementioned evidence, it is reasonable to infer that the beneficial effects of vinpocetine are associated with its capacity to elevate levels of second messengers and transcription factors, thereby counteracting impaired catecholaminergic transmission. It should be noted that both cAMP/PKG and cGMP/PKG signaling systems are involved in controlling several cellular processes related to metabolism, gene transcription, and neurotransmission [81]. Thus, it is difficult to identify the mechanism(s) connecting these cascades and lead-induced hyperactivity.

Besides PDE1 inhibition, vinpocetine could have other actions that play a role in ameliorating hyperactivity in lead-exposed mice. For instance, in rats prenatally exposed to ethanol, vinpocetine promotes significant anti-inflammatory action with the reduction in the levels of pro-inflammatory cytokines tumor necrosis factor-alpha (TNF-α) and interleukin 6 (IL-6), along with the increase in the levels of the anti-inflammatory interleukin-10 (IL-10) [27]. In the same study, it was observed that vinpocetine (10 and 20 mg/kg) resulted in a decrease in the TBARS levels and an increase in the levels of the antioxidant molecules reduced glutathione (GSH) and superoxide dismutase (SOD) in various regions, indicating a reduction in oxidative stress promoted by ethanol [27]. It is important to note that lead exposure promotes both inflammation and oxidative stress in brain tissue [82]. Recently, the endogenous antioxidant system and neuroinflammation have been implicated in the pathophysiology of ADHD [83,84].

### 4.3. Study Limitations and Perspectives

A potential limitation of our study was the lack of long-term behavioral assessments. Even though the molecular mechanism involved in long-lasting changes in synaptic efficiency has been associated with the activation of both CREB and SRF [85,86], the effects of vinpocetine as a restorative agent have only been confirmed for short periods after treatment [21,28,31]. Therefore, the investigation of whether the improvement in lead-induced neurobehavioral deficits is long-lasting remains to be carried out. Considering that the effects of vinpocetine may not last for more than a few hours after a single injection [28,87], the use of multiple injections should be taken into account. In addition, a more extensive study could fully address the effects of vinpocetine in other neurobehavioral disorders that have been associated with developmental lead exposure such as learning and memory deficits and autistic behavior.

It is noteworthy that developmental lead exposure has been linked not only to behavioral symptoms of ADHD but also to schizophrenia [4], lower intelligence quotient [88], impairments in learning and memory [89], and autistic behavior [90]. This large spectrum of neurobehavioral disorders might be associated with the plural nature of the lead insult and, in turn, constrains the development of therapeutic approaches for neurobehavioral problems observed in subjects early exposed to lead. In this regard, the use of vinpocetine might be interesting. In animal models of fetal alcohol spectrum disorder, vinpocetine treatment was able to mitigate locomotor hyperactivity, inattention [27], anxiety, and learning memory deficits [21,27,31]. Vinpocetine was also shown to have dose-dependent anticonvulsant effects [91], was able to improve dyskinesia in a rat model of Parkinson disease [92], and rectified behavioral phenotypes associated with autism in rats [93]. Vinpocetine has already been tested in humans as a neuroprotective agent in acute ischemic stroke [94] and as a cognitive enhancer in healthy volunteers and in patients with epilepsy [87].

## 5. Conclusions

The absence of locomotor hyperactivity in vinpocetine-treated female mice exposed to lead reinforces the idea that impairments in the cAMP and cGMP signaling systems may underlie lead-induced hyperactivity. Since vinpocetine treatment was administered long after the lead exposure period, during a stage comparable to the onset of adolescence in humans, our findings may have clinical relevance, as they suggest the possibility of treating hyperactivity in juveniles when prevention fails. New studies addressing different time periods of treatment and/or behavioral endpoints could yield valuable information in this regard.

## Figures and Tables

**Table 1:** Blood lead levels (µg/dL).

Experimental Groups	PN10	PN30
CONT	0.8 ± 0.2 (0.6 to 1.1)	1.2 ± 0.2 (0.6 to 2.2)
LEAD	28.9 ± 4.0 (12.5 to 47.0) ***	2.1 ± 0.8 (0.6 to 6.1)

Values are mean ± SEM. FPLSD: *** *p* < 0.001 in comparison with all other groups.

## Data Availability

The raw data supporting the conclusions of this article are included in the article; further inquiries can be directed to the corresponding author.
